# Potential Metabolomic Linkage in Blood between Parkinson’s Disease and Traumatic Brain Injury

**DOI:** 10.3390/metabo8030050

**Published:** 2018-09-07

**Authors:** Massimo S. Fiandaca, Thomas J. Gross, Thomas M. Johnson, Michele T. Hu, Samuel Evetts, Richard Wade-Martins, Kian Merchant-Borna, Jeffrey Bazarian, Amrita K. Cheema, Mark Mapstone, Howard J. Federoff

**Affiliations:** 1 Translational Laboratory and Biorepository, Department of Neurology, University of California Irvine School of Medicine, Irvine, CA 92697-3910, USA; tjgross@uci.edu (T.J.G.); mark.mapstone@uci.edu (M.M.); 2 Department of Neurological Surgery, University of California Irvine School of Medicine, Irvine, CA 92697-3910, USA; 3 Department of Anatomy & Neurobiology, University of California Irvine School of Medicine, Irvine, CA 92697-3910, USA; 4 Intrepid Spirit Concussion Recovery Center, Naval Medical Center Camp Lejeune, Jacksonville, NC 28540, USA; thomas.m.johnson74.mil@mail.mil; 5 Nuffield Department of Clinical Neurosciences, University of Oxford, 01865 Oxford, UK; michele.hu@ndcn.ox.ac.uk (M.T.H.); samuel.evetts@ndcn.ox.ac.uk (S.E.); 6 Department of Neurology, John Radcliffe Hospital, Oxford University Hospitals Trust, Oxford 01865, UK; 7 Department of Physiology, Anatomy and Genetics, Oxford Parkinson’s Disease Centre, University of Oxford, Oxford 01865, UK; richard.wade-martins@dpag.ox.ac.uk; 8 Department of Emergency Medicine, University of Rochester School of Medicine and Dentistry, Rochester, NY 14604, USA; Kian_Merchant-Borna@URMC.Rochester.edu (K.M.-B.); jeff_bazarian@urmc.rochester.edu (J.B.); 9 Department of Oncology, Lombardi Comprehensive Cancer Center, Georgetown University Medical Center, Washington, DC 20001, USA; amrita.cheema@georgetown.edu; 10 Department of Biochemistry and Molecular & Cellular Biology, Georgetown University Medical Center, Washington, DC 20001, USA

**Keywords:** Parkinson’s disease, Parkinson’s disease dementia, subacute mild traumatic brain injury, glutamic acid, excitotoxicity, metabolomics

## Abstract

The etiologic basis for sporadic forms of neurodegenerative diseases has been elusive but likely represents the product of genetic predisposition and various environmental factors. Specific gene-environment interactions have become more salient owing, in part, to the elucidation of epigenetic mechanisms and their impact on health and disease. The linkage between traumatic brain injury (TBI) and Parkinson’s disease (PD) is one such association that currently lacks a mechanistic basis. Herein, we present preliminary blood-based metabolomic evidence in support of potential association between TBI and PD. Using untargeted and targeted high-performance liquid chromatography-mass spectrometry we identified metabolomic biomarker profiles in a cohort of symptomatic mild TBI (mTBI) subjects (*n* = 75) 3–12 months following injury (subacute) and TBI controls (*n* = 20), and a PD cohort with known PD (*n* = 20) or PD dementia (PDD) (*n* = 20) and PD controls (*n* = 20). Surprisingly, blood glutamic acid levels in both the subacute mTBI (increased) and PD/PDD (decreased) groups were notably altered from control levels. The observed changes in blood glutamic acid levels in mTBI and PD/PDD are discussed in relation to other metabolite profiling studies. Should our preliminary results be replicated in comparable metabolomic investigations of TBI and PD cohorts, they may contribute to an “excitotoxic” linkage between TBI and PD/PDD.

## 1. Introduction

Compelling epidemiological observations associate moderate and severe traumatic brain injury (TBI) and Parkinson’s disease (PD) [1]. Whether mild TBI (mTBI) is a significant risk factor for the development of PD (and other neurodegenerative disorders) has been more difficult to prove, due to fewer controlled investigations [2,3,4], conflicting results [5], and a lack of agreement on diagnostic criteria [6]. We anticipate that molecular phenotyping may ultimately resolve the latter discrepancies in the definition of mTBI. Recent studies [7,8], however, have more strongly endorsed an association between PD and TBI (including mTBI) sustained both early or later in life. Absent a consensus regarding a potential post-traumatic etiology for PD (or dementing conditions), the future definition of such relationships likely requires comprehensive longitudinal investigations and novel biomarkers [9]. Despite the limitations in current knowledge, there is emerging agreement that chronic neuroinflammatory conditions are associated with clinical parkinsonism and/or dementia, if not true PD or Alzheimer’s disease (AD), and significant pathobiologic overlap exists (i.e., neuroinflammation, oxidative stress response, mitochondrial dysfunction, cognitive decline, and clinical depression) between neurodegenerative disorders (e.g., AD and D) and TBI [10,11]. The mechanisms underlying a precipitating event such as TBI to those downstream dysregulated networks associated with neurodegenerative diseases remains unknown.

For this article, as well as our previous report on acute mild brain trauma biomarkers [12], we based our diagnosis of mTBI (including the term concussion) on diagnostic criteria provided by our medical co-authors and medical doctors involved in the assessment of study participants. We have reported a set of human plasma metabolites associated with acute mTBI (within 6 h of injury) that accurately classify concussed individuals from non-concussed controls [12]. In this extension of our mTBI biomarker efforts we sought to define metabolomic similarities and differences between plasma specimens from a subacute cohort that includes subjects 3 to 12 months following mTBI, the previously reported acute mTBI biomarker panel, and in a cross-sectional design, whether plasma metabolites with TBI provide novel insights related to potential future risk of PD.

## 2. Results 

### 2.1. Study Population Differences

A comparison of the demographics for the study cohorts is provided in Table 1. Our TBI cohort consisted of 75 cases and 20 controls. Described values are provided as the mean and standard deviation (S.D.). Frequency distribution of ages for the cases and controls in the TBI cohort did not follow a normal distribution, while ages in the PD cohort did. The TBI cases had a mean age of 24.9 ± 5.2 years, with 71 males and 4 females represented, and all of whom sustained a TBI during a three to twelve month interval prior to phlebotomy. The TBI controls (*n =* 20) had a mean age of 18.7 ± 0.8 years, included 8 males and 12 females, and did not have a history of a witnessed concussion or mTBI during the previous year prior to blood draw. Statistically significant age and sex differences existed between cases and controls in the TBI cohort. All TBI case and control participants attained the minimum of a high school graduate level of education. The number of injuries sustained by the TBI cases ranged from 1 to 9, with a mean of 2.0 ± 1.5. The severity of the last medically documented injury was a mTBI or concussion in 71 cases and moderate TBI in the other 4 cases. Individuals with TBIs prior to the last one reported injuries 12 months to 11 years prior, with a mean of 3.8 ± 3.7 years. Subjects in the PD cohort (*n =* 60) consisted of the PD (*n =* 20) and PD dementia (PDD) (*n =* 20) cases (combined *n =* 40), and the PD controls (*n =* 20). The PD cohort was approximately 40 years older than the TBI cohort. Mean ages ( ±S.D.) for the PD cohort, as well as the PD/PDD, PD, PDD, and PD control groups were 66.8 ± 11.0, 67.2 ± 11.4, 62.9 ± 10.4, 71.6 ± 10.9, and 65.9 ± 10.3 years, respectively. The mean age of the PD/PDD cases and the PD controls were not significantly different. Commensurate with previous studies, a male to female preponderance was noted across the PD cohort (overall 33 males and 27 females, with 22 males and 18 females making up the PD/PDD cases, and 11 males and 9 females being PD controls). There were no statistically significant sex differences between cases and controls in the PD cohort. At the time of blood collections on average, PD and PDD subjects were 2.9 ± 1.2 and 3.4 ± 1.2 years, respectively, from their original PD diagnosis. The TBI cohort subjects provided plasma for metabolomic analysis, while the PD cohort subjects provided serum. 

### 2.2. Subacute mTBI Plasma Metabolomic Biomarkers–MetaboAnalyst 4.0 Method

Of the top 15 preliminarily annotated metabolites derived using each of the unbiased feature selection algorithms within the Explorer module of MetaboAnalyst 4.0, the top nine are presented in Table 2, along with their qualitative differences between controls and cases. The metabolites are designated by their preliminarily annotated names followed by an appropriate structural symbol (as required) and finally a letter designation of whether identified in (N)egative or (P)ositive electrospray ionization (ESI) mode. Three of the top 9 metabolites (denoted by asterisk) were common to each of the four possible unbiased feature selection methods available. Of the nine, six specific metabolites combined in a classification model provided highly accurate receiver operating characteristic area under the curve (ROC AUC) results for distinguishing control subjects from those with subacute mTBI (Table 3). This 6-member panel provided classification AUCs of ≥0.9 for each of the analytic methods evaluated. Similar classification ROC AUC results were obtained using least absolute shrinkage and selection operator (LASSO) feature selection and a disparate group of 9 of the top 10 metabolites (data not shown), that also excluded the top-ranked Monoacylglycerol (MG) C16:0_N, but did include Creatinine_N and Glutamic Acid_N. Inclusion of MG C16:0_N alone, or in combination with other metabolites, provided ROC AUC values approaching 1.0, but did not allow model convergence required to provide ROC AUC and sensitivity and specificity results associated with the LR + 10FCV algorithm within MetaboAnalyst 4.0. 

### 2.3. Subacute Plasma mTBI Metabolomic Biomarkers–mixOmics, sPLS-DA Method

**Figure 1:**
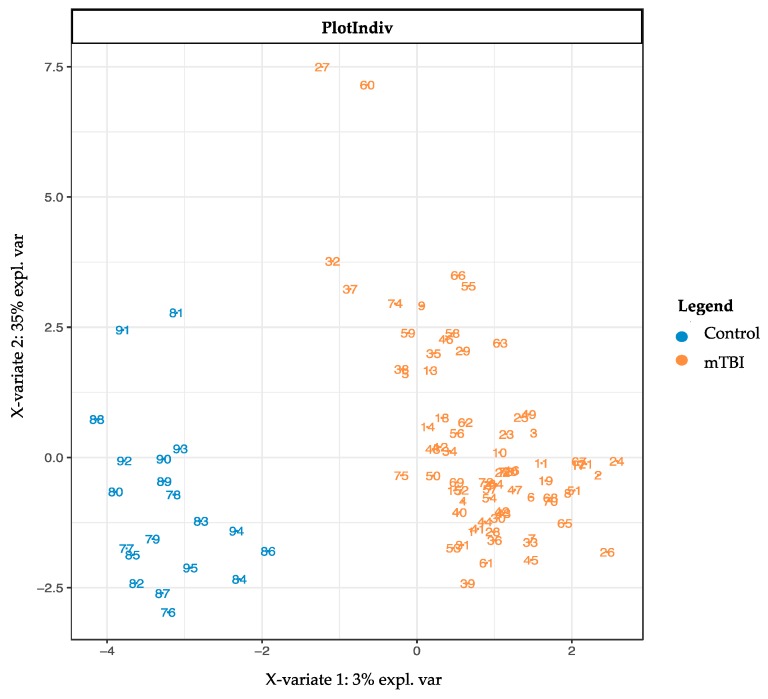
Sparse partial least squares discriminant analysis (sPLS-DA) plot. Note separation of subacute mTBI compared to TBI control data, as determined by metabolites making up the first two analytic components. The separation of the case and control groups is complete, without overlap. sPLS-DA = sparse partial least squares-discriminant analysis. Control = TBI control. mTBI = mild traumatic brain injury.

**Figure 2:**
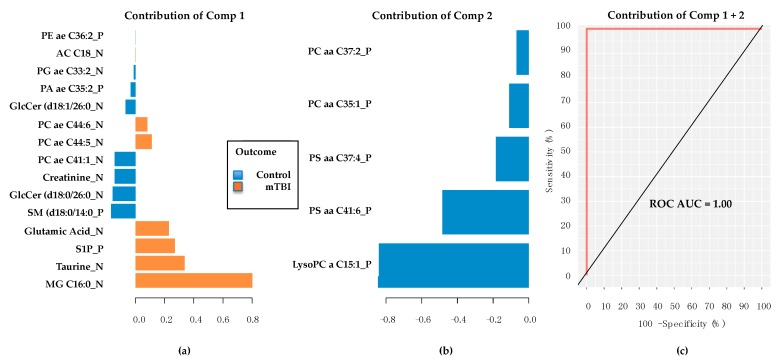
Metabolites associated with first two discriminant components. (**a**) The first component provides 15 metabolites, and the bottom 4 listed providing the greatest contributions (all higher in TBI cases) to classification accuracy. (**b**) The second principal component provides 5 metabolites (all lower in TBI controls). (**c**) Receiver operating characteristic area under the curve (ROC AUC) provides result of 1.00 using 20 metabolites from the two components in the classifier model. Comp = sPLS-DA model component. PE = phosphatidylethanolamine. AC = acylcarnitine. PG = Phosphatidylglycerol. PA = Phosphatidic acid. GlcCer = glucosylceramide. PC = phosphatidylcholine. SM = sphingomyelin. S1P = sphingosine-1-phosphate. MG = Monoacylglycerol. PS = phosphatidylserine. LysoPC = lysophosphatidylserine. Final metabolite identifications will require additional tandem mass spectrometry (MS/MS) analyses. Metabolites confirmed via MS/MS are considered fully validated, to a high degree of confidence.

The subacute mTBI cases and controls could readily be distinguished using graphical sparse partial least squares discriminant analysis (sPLS-DA) plots (Figure 1) within *mixOmics*, showing a complete group separation on the two component axes. Ten repetitions of 10-fold cross validation provided a final sPLS-DA 2 component model that provided error-free classification via 20 metabolites (Figure 2) that included the most significant Monoacylglycerol C16:0_N, which was excluded from all the convergent MetaboAnalyst 4.0-derived results.

### 2.4. Subacute mTBI Plasma Metabolomic Biomarkers–Targeted Analysis via mixOmics

**Figure 3:**
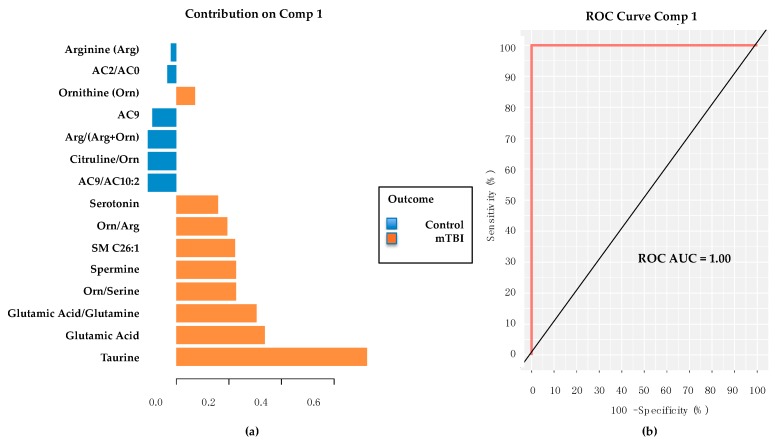
Targeted metabolomic panel and classification performance. Using the sPLS-DA methods in *mixOmics*, this 15-member metabolite panel was derived (**a**) featuring primarily amino acids, biogenic amines and specific metabolite ratios. This particular targeted metabolite panel classified subacute mTBI subjects from TBI controls with a ROC AUC = 1.0. (**b**) Note the two metabolites with the highest contribution are Taurine and Glutamic Acid. Comp 1 = feature selection component 1. mTBI = mild traumatic brain injury. ROC = receiver operating characteristic. AUC = area under the curve. AC = acylcarnitine. SM = sphingomyelin.

Targeted metabolite (Biocrates AbsoluteIDQ® p180 kit, Biocrates Life Sciences AG, Innsbruck, Austria) values were developed into an optimal classification model using 10 repetitions of 10-fold cross validation through sPLS-DA in *mixOmics*. The final model featured 15 metabolites and metabolite ratios (Figure 3a) that provided perfect classification of the groups (Figure 3b). Of interest, both Taurine and Glutamic Acid were top contributors to the panel, thereby indirectly supporting their putative identities and importance derived from the untargeted analyses previously presented, with both elevated in the subacute mTBI cases, as opposed to controls. 

In summary, we discovered and internally validated several plasma metabolomic biomarker panels using both untargeted and targeted metabolomic approaches and using two different analytic platforms, MetaboAnalyst 4.0 and *mixOmics*. The final biomarker panels derived by the untargeted methods featured several of the same top metabolites as the targeted analysis, and suggested potential relevance for both Glutamic Acid and Taurine in subacute TBI. Of interest, the top 4 metabolites resulting from unbiased feature selection via MetaboAnalyst 4.0 and *mixOmics* were identical (see Table 2 and Figure 2a). Additional investigations are required to confirm the identification of the preliminarily annotated plasma biomarkers proposed in this study using untargeted methods. While tandem MS (MS/MS) is typically required, a preliminary confirmation of both Taurine and Glutamic Acid can be proposed given the confirmed identities provided by the targeted metabolomic results. It remains important, however, that the preliminarily annotated plasma metabolomic panels for subacute mTBI be externally replicated utilizing similar groups of cases and controls.

### 2.5. PD/PDD Serum Metabolomic Biomarkers–Utilizing the mixOmics-Derived sPLS-DA Top 20 Metabolites from Subacute mTBI Analysis 

**Figure 4:**
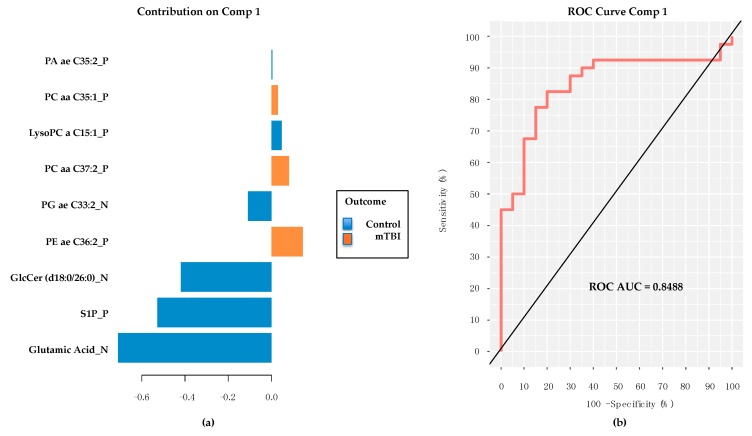
Contribution plot and performance of 9 common subacute TBI biomarkers classifying the PD/PPD subjects from PD controls. (**a**) Note prominence of the Glutamic Acid contribution, but with relative abundance values reduced in PD/PDD and compared to controls. (**b**) Respectable performance (ROC AUC *=* 0.8488) of 9 member panel in classifying PD/PDD subjects from controls. Comp *=* PLS-DA model component. TBI *=* traumatic brain injury. PD *=* Parkinson’s disease. PDD *=* PD dementia. ROC AUC *=* receiver operating characteristic area under the curve. PA *=* Phosphatidic acid. PC *=* phosphatidylcholine. LysoPC lysophosphatidylcholine. PG *=* Phosphatidylglycerol. PE *=* phosphatidylethanolamine. GlcCer *=* glucosylceramide. S1P *=* sphingosine-1-phosphate. Final metabolite identifications will require additional tandem mass spectrometry (MS/MS) analyses. Metabolites confirmed via MS/MS are considered fully validated, to a high degree of confidence.

Metabolite matching using *MSFmetabolomics*, between the 20 sPLS-DA-derived plasma subacute mTBI metabolite biomarkers and the serum-derived PD/PDD/Control metabolomic data, indicated that only nine of the 20 metabolites were also present in preliminarily annotated metabolites from the PD/PDD/Control specimens (Figure 4a). Despite such a limitation in numbers of matched metabolites between the two datasets, the performance of the 9-metabolite panel in a *mixOmics* PLS-DA classifier model provided respectable ROC AUC (0.8488) results (Figure 4b). Importantly, Glutamic Acid was again a prominent contributor to the model’s performance, although this time it was notably increased in control subjects in comparison to the PD/PDD group. Taurine was not present as a member of this panel. These findings suggest a relative loss of serum Glutamic Acid concentration in those with PD/PDD compared to age-matched controls, while the absence of Taurine from the panel likely represents an insignificant difference in levels between PD/PDD and control subjects.

Utilizing the subacute mTBI metabolite panel members in a group of much older PD/PDD/Control subjects provided very good classification accuracy for discriminating PD/PPD from matched controls, and despite using only 9 of the original 20 metabolites in the model. Although encouraging, these findings are limited by the relatively small group sizes in the PD/PDD/Control cohort, with only 20 individuals represented in each diagnostic category. Larger numbers of subjects may provide alternative impressions, as well as analyzing the PD cohort’s plasma specimens rather than serum. Impressively, however, Glutamic Acid remained the most significant metabolite differentiating cases from controls in the PD cohort analysis, with the opposite relative abundance (higher in controls rather than cases) to that found in the subacute mTBI subjects.

### 2.6. PD/PDD Serum Metabolomic Biomarkers–New Discovery Using mixOmics sPLS-DA 

**Figure 5:**
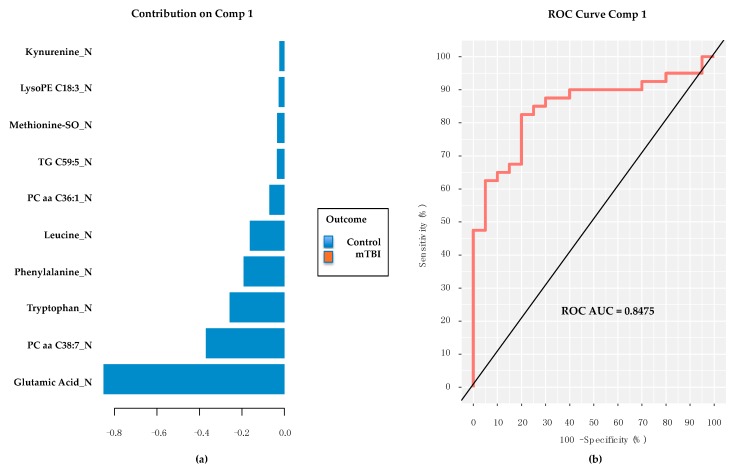
Contribution plot and classification performance of 10 metabolites derived via sPLS-DA from PD/PPD/Control subjects. (**a**) Glutamic Acid continues to provide the major contribution to the classification performance of this preliminarily annotated 10-metabolite panel. (**b**) A similar ROC AUC is obtained in new discovery with these data as had been obtained using the subacute TBI biomarker panel’s 9 preliminarily annotated common metabolites (see Figure 4). Of interest, the only common metabolite between these results and those from the TBI panel is Glutamic Acid. Comp = sPLS-DA model component. TBI = traumatic brain injury. PD = Parkinson’s disease. PDD = PD dementia. ROC AUC = receiver operating characteristic area under the curve. LysoPE = lysophosphatidylethanolamine. SO = sulfoxide. TG = triglyceride. P = phosphatidylcholine. Final metabolite identifications will require additional tandem mass spectrometry (MS/MS) analyses. Metabolites confirmed via MS/MS are considered fully validated, to a high degree of confidence.

Utilizing the *mixOmics* platform and sPLS-DA, unbiased feature selection was used to discover an optimal classification model when comparing the PD/PDD group to PD controls. Using 10 repetitions of 10-fold cross-validation a model utilizing a single component composed of 10 metabolites was developed (Figure 5a). The model’s classification contribution was significantly weighted toward Glutamic Acid, which was again higher in the serum of control subjects than in those with PD/PDD. As in the previous section, performance of this 10 member panel provided an ROC AUC of 0.85 (Figure 5b).

### 2.7. Evaluation of Glutamic Acid’s Performance as Sole Metabolite in mixOmics PLS-DA Classifier Models for Subacute mTBI and PD Cohorts 

**Figure 6:**
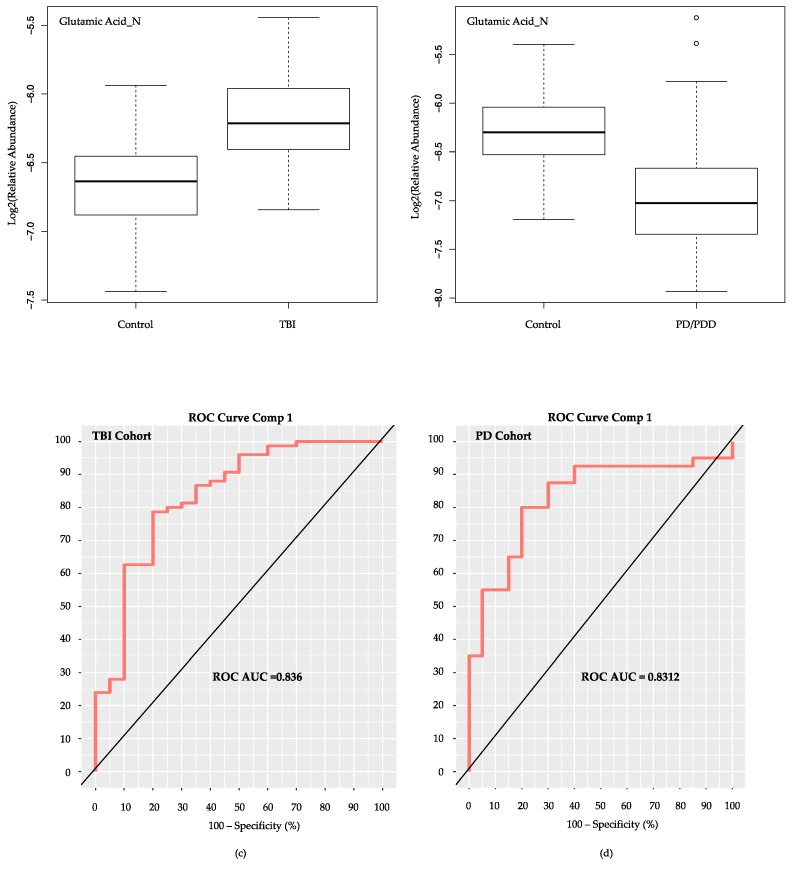
Classification of cohort groups using Glutamic Acid as the sole metabolite. Log2 (relative abundance) values for Glutamic Acid in the two study cohorts are depicted via boxplots in panels (**a**,**b**). For the subacute TBI cohort (**a**) Glutamic Acid is elevated in TBI cases compared to controls. In the PD cohort (**b**) Glutamic Acid was elevated in controls compared to the PD/PDD cases. ROC AUC results are nearly identical using the *mixOmics* PLS-DA model with only Glutamic Acid as classifier of the TBI (subacute mTBI) cases from TBI controls (**c**), as well as the PD (PD/PDD) cases from PD controls (**d**). Comp = PLS-DA model component. TBI = traumatic brain injury. mTBI = mild TBI. PD = Parkinson’s disease. PDD = PD dementia. ROC AUC = receiver operating characteristic area under the curve.

Relative abundance values for Glutamic Acid were higher in the TBI cases as opposed to TBI controls (Figure 6a), while controls provided higher abundance values than cases in the PD cohort (Figure 6b) We tested the classification ability of Glutamic Acid as a sole classifier for both of our cohorts, the subacute mTBI and PD. Using the *mixOmics* PLS-DA algorithm, and Glutamic Acid alone, comparable classification ROC AUC results were attained in both cohorts (Figure 6c,d), despite the opposite relative abundance measures noted between cases and controls. 

## 3. Discussion 

In addition to the prominence of Taurine and Glutamic Acid in blood specimens from our subacute mTBI subjects, elevations of the Glutamic Acid/Glutamine ratio in the targeted metabolomic results suggests potential alterations in the cycling of these two species during the subacute recovery from TBI. Such an altered ratio has been previously noted in both children and adults following acute TBI [13,14]. Interestingly, Taurine is known to function as an osmoregulator [15], neuromodulator, calcium regulator [16], antioxidant [17], and neuroprotectant from excitotoxic cell death [18]. It does not require much extrapolation, therefore, to see how these two metabolites may be integral responses to continuing, subacute processes in response to a TBI. 

Glutamic acid, or glutamate is the most abundant excitatory neurotransmitter in brain tissue [19,20]. Physiologically, glutamate helps mediate cellular function through binding to glutamate receptor (GluR) proteins localized to the external face of the plasma membrane [21] and thereby activating a variety of ion channels or intracellular networks via G-proteins and other membrane and cytoplasmic mediators. Levels of glutamate in the brain’s extracellular fluid (ECF) are typically maintained within a tight range, and thus, concentrations that are too low or too high may produce negative consequences [20]. Regulation of glutamate levels in the brain ECF, therefore, is important in preventing cellular toxicity. Synthesis of brain glutamate involves uptake of peripherally derived branched chain amino acids (BCAA) from blood, with their uptake and intracellular processing to form glutamine taking place within astrocytes, and release of glutamine from astrocytes and uptake by neurons finally resulting in production of glutamate from glutamine, and glutamate’s eventual release as a neurotransmitter [22]. Since there are no glutamate degrading enzymes within the ECF, regulation of glutamate levels is controlled via cellular release and cellular uptake. The primary mechanism that controls the brain’s ECF glutamate levels under normal physiological conditions is via uptake/transport mechanisms associated with local neurons, astroglia, and the endothelial cell components of the blood-brain barrier (BBB) [20,23]. Specific abluminal transporters on brain vascular endothelium vessels and within the choroid plexus aid in regulating ECF and cerebrospinal fluid (CSF) glutamate levels by transport of excess glutamate into endothelial cells of the neurovasculature and thereby released into circulating blood [24,25]. In contrast, direct uptake of glutamate from blood is insignificant [25,26,27]. In known cases of toxic glutamate levels in brain, such following ischemic stroke or TBI, such unidirectional flux may potentially be modulated to help restore homeostasis [28] and improve outcomes. In TBI, glutamate is known to increase acutely within the ECF as a result of the associated cellular injury and BBB dysfunction/disruption, with abnormalities in the physiologic uptake/transport mechanisms [29]. While there is robust evidence for changes in ECF glutamate following acute TBI [13,14,30,31], similar analyses during the subacute stage of TBI recovery are limited. Our current analyses suggest that glutamate might remain increased in plasma for at least 3–12 months following mTBI, at least in those symptomatic individuals within our subacute mTBI group. We speculate that elevated levels of Taurine might be an attempt at physiological buffering of what might otherwise be considered a relatively “excitotoxic” environment [32] if elevated Glutamic Acid levels in plasma reflect similar conditions in the brain ECF of the subacute mTBI subjects. Finally, we propose that glutamate elevations in brain ECF may be a direct expression of the degree of parenchymal injury sustained, while elevations in Taurine may indicate an intrinsic attempt to mitigate progressive secondary injury effects. 

Concussion (or mTBI) produces early ECF increases in free fatty acids [33], via activation of phospholipases [34]. Elevations in the free saturated fatty acids (SFA) palmitate and stearate rapidly increase in brain following experimental TBI, achieve concentrations 2–3 times those of the polyunsaturated fatty acid arachidonic acid, and remain elevated beyond 6 h following injury [35]. Release of membrane SFAs is the result of phospholipase A_1_ (PLA_1_) activity on the plasma membrane [36,37]. We have recently been able to link levels of palmitate to dysregulated expression of a bioenergetic regulator in PD [38], which may ultimately prove significant in other neurological conditions, including TBI. Both PD [39] and AD [40] are characterized by reductions in brain levels of the peroxisome proliferator-activated receptor gamma coactivator 1-alpha (PGC-1α), a major regulator of mitochondrial number and function, also known to control cellular lipid metabolism, glucose metabolism, electron transport, and certain anti-inflammatory effects. We have shown that elevated levels of the free palmitate reduce PGC-1α gene expression through the epigenetic non-canonical promoter hypermethylation, both in vitro and in vivo [38]. The relation of cellular PGC-1α levels to TBI does not appear to be as straightforward as in PD, but may result from the previously noted elevations in palmitate following brain trauma and secondary effects that maintain abnormally elevated free palmitate levels. Whether elevated levels of palmitate result in additional complex reactions [41] impacting brain parenchyma remains to be determined. Importantly, however, a link between abnormal lipid levels in brain (and likely blood) together with downregulation of PGC-1α gene expression have been made in PD [42] and AD [43], and appear related to metabolism and epigenetic controls on gene expression. Whether such links can be made in TBI remains to be seen.

As opposed to TBI, the susceptibility of brain to glutamate toxicity has been primarily demonstrated following hypoxic/ischemic insults [44,45], commonly associated with excessive increases in measured ECF glutamate levels. Although similar elevations in ECF glutamate levels have been associated with severe TBI, significant elevations in mTBI (i.e., concussions) or neurodegenerative disorders (e.g., PD or AD) have been considered less likely [46]. Much more likely, however, is that in PD and other neurodegenerative disorders, and in more diminished brain insults (e.g., mTBI), less dramatic ECF glutamate levels may somehow become toxic and perpetuate a cellular injury cascade. Varying susceptibilities to toxicity from physiological glutamate levels may involve mitochondrial energy metabolism, and the energy-dependent maintenance of neuronal membrane polarization. Since energy-dependent ion channels and pumps are primarily responsible for sustaining the resting membrane potential of neurons, a depletion of adenosine triphosphate (ATP), associated with mitochondrial dysfunction (as in PD, accompanied by reduced PGC-1α expression), will result in a reduced membrane potential or actual depolarization [47]. Alternative hypotheses for glutamate cytotoxicity in neurodegenerative disorders have been proposed [48,49] and provide a much more solid experimental foundation [50]. These postulates highlight the synergistic interaction [51] between bioenergetic defects and glutamate toxicity at physiologic levels.

The Glutamic Acid and Taurine elevations seen in our subacute mTBI cases were absent in the PD/PDD cases assessed. Subjects with PD (and PDD) likely suffer from varying degrees of brain mitochondrial dysfunction, featuring aberrant lipid and glucose metabolism, and altered energy production as a result of epigenetic downregulation of PGC-1α, among other mechanisms [38]. While such susceptibilities in PD may be limited to modulation of subcortical motor pathways, resulting from deficits related to dopaminergic nigrostriatal degeneration, as the pathobiology progresses to include PDD, the susceptible brain regions may expand to involve cortical gray matter neuronal populations critical to higher order cognition. Metabolite profiling in PD remains challenging, with the most common findings related to alterations associated with mitochondrial dysfunction [52]. In this analysis between age-matched controls and PD/PDD cases, we found relative elevations in serum Glutamic Acid levels in the control subjects compared to cases, as previously noted using nuclear magnetic resonance (NMR) analyses of in vitro PD models [53] and CSF in human PD [54], along with PD plasma using similar MS methods to our own [55]. Such results, however, contradict prior blood-based results using less sensitive methodologies [56]. Our Glutamic Acid findings in PD/PDD serum are supported by our use of UPLC-MS, and may reflect a pathological reduction of brain ECF glutamate levels, a relative exhaustion of glutamate production in PD/PDD, or an attempted compensatory reduction of serum Glutamic Acid levels (resulting from reduced excitatory neurotransmitter tone within the brain) reducing the susceptibility of “excitotoxicity”. 

We acknowledge specific limitations associated with our biomarker investigation. A common specimen collection and processing protocol for both cohorts would have been ideal, but were not possible for this study. Given our past experience collecting, processing, and analyzing metabolomic specimens, we only accepted and analyzed specimens that we felt met strict collection and processing standards. We acknowledge, however, that differences in whether the specimens were collected fasting or not, and processed within 4 or 24 h from collection, to produce either plasma or serum, may have adversely impacted our ability to adequately interpret the results. Measuring the oxidation of lipids, especially phosphatidylcholines (PCs) in biospecimens, as a determinant of specimen integrity or enhanced disease-related phospholipase activity, have been reported in humans [57,58,59]. In relation to AD biospecimens [57,58], the ratio of Lysophosphatidylcholines (LysoPCs)/PCs has been proposed as useful in differentiating between control subjects and those with prodromal or manifest disease, possibly reflecting pathologic membrane oxidation. Such ratios can more accurately be determined using quantitative targeted MS results, with such ratios increasingly provided in analytic outputs. Although the metabolomic field, to our knowledge, has yet to adopt routine use of such ratios as determinants of specimen integrity, we are in support of an eventual consensus measure that would allow discrimination of specimen integrity [60]. For this investigation, comparison of targeted metabolomic output results, and calculation of LysoPC/PC ratios was not possible between our TBI and PD cohort specimens, since targeted analyses were only available from the former. Despite an adequate number of available subacute mTBI specimens, we admit including a less than optimally matched set of TBI control group specimens, both in number and comparable characteristics. As presented in Table 1 and described in Results 2.1, there were significant age and sex differences noted within the TBI cases compared to TBI control groups. These dissimilarities likely resulted from the inclusion of subjects from two separate, independent investigations in our study, with one featuring military personnel and the other made up of college athletes. Ideally a military non-TBI cohort from the same military institution as the TBI cases would have likely provided a better-matched control group for the subacute mTBI cases. Additional TBI controls would have strengthened the analysis as well, especially with the inclusion of a number of non-TBI, trauma controls (e.g., orthopedic injuries), to attempt differentiation of TBI-specific biomarkers from those related to a more generalized post-traumatic state [61]. It remains possible that the age and sex differences between the mTBI cases and controls may have somehow contributed to the observed metabolomic differences. Although the PD cohort’s groups were much better balanced and matched on all parameters, we believe that larger numbers in each of the subgroups could provide added weight to the results. The addition of subjects from the preclinical PD spectrum, including those diagnosed with rapid eye movement (REM) sleep behavior disorder (RBD) [62,63,64] without PD, considered a preclinical non-motor stage of PD, might have allowed blood-based Glutamic Acid assessment during this transition to the clinically evident motor stages of PD. Our goal for future investigations assessing biomarkers in both TBI and PD will include evaluating larger, more comparable cohorts of subjects (including matching ages and sex in cases and controls) and specimens (using the same collection protocols and blood matrix for analysis). Increased detail should be paid to lifelong histories of TBI experiences, the ethnicity of participants, environmental exposures (e.g., rural versus urban living), and mitigating any geographical bias between groups. While an initial homogeneity of cohorts might be helpful in defining significant classifiers of specific conditions, once such classifiers are determined and replicated under the same settings, stress testing of such panels in more disparate subject groups would be a requisite next step toward biomarker development and more widespread utility. Finally, we believe it is important to avoid analyses of disparate blood matrices whenever possible. For this study we did not have the option to evaluate only plasma or serum within both cohorts, as the TBI cohort only provided plasma while the PD cohort had collected serum. Such comparisons, we believe, are not as ideal, as we have raised previously [60]. Evidence of differences between serum and plasma metabolites within the same subjects has been documented [65], and such differences are especially notable for certain glycerophospholipids [66]. Despite these limitations, we believe the information developed through this study provides relevant preliminary guidance related to potential pathobiologic links between subacute mTBI and PD, with the prominence of Glutamic Acid in blood, or lack thereof, in both cohorts. Replicative investigations are necessary to assess the significance, if any, of blood Glutamic Acid as a biomarker for subacute mTBI, and possibly for staging PD. Such investigations will detail whether there are consequential ties between mTBI and PD. 

## 4. Materials and Methods 

### 4.1. Study Populations

The institutional review board (IRB) at Naval Medical Center Portsmouth, VA, approved study protocol, informed consent documents, and participation for all consenting subacute mTBI participants from Naval Medical Center Camp Lejeune in compliance with all applicable Federal regulations governing the protection of human subjects. The Research Subjects Review Board at the University of Rochester and Rochester Institute of Technology provided approval for human subject participation for the TBI control participants, all of whom provided written informed consent prior to entering the study [67] and providing specimens. Control, PD, and PDD subjects giving informed consent for study participation and collection of blood specimens were part of an Oxford Parkinson’s Disease Centre (OPDC) Discovery Cohort study, Oxford, UK approved by the Oxfordshire A Research Ethics Committee (10/H0505/71, Version 5, 23/07/14), with transfer of specimens to Georgetown University and University of California Irvine (UCI) approved by the OPDC Data Access Committee. In addition, all protocols, consents, and relevant documents for each individual study and combined storage and analyses of de-identified specimens and study protocols were approved by the IRBs at Georgetown University and the University of California, Irvine, and by the Department of Defense Human Research Protection Office. 

The subacute mTBI cohort was made up of 75 active duty sailors and marines cared for at the Intrepid Spirit Concussion Recovery Center, Naval Medical Center Camp Lejeune, Jacksonville, NC. All study participants had sustained a TBI within the 3–12 month interval (subacute period of recovery) prior to blood collection, and all were being followed for persistent neuropsychological symptoms following their TBIs. Control TBI subjects (*n* = 20) were asymptomatic, non-concussed collegiate athletes participating in an acute sports-related mTBI study [67] in Rochester, NY. Included athletes for this study provided blood specimens prior to their participation in their respective sports season and had no history of a recent TBI (within the previous 12 months). PD subjects were recruited from the longitudinally assessed, population-ascertained Oxford Discovery Cohort [68]. The clinical diagnosis of PD and PDD was made according to UK PD Society Brain Bank diagnostic criteria [69], and Movement Disorders Society level 1 criteria for PDD [70] during 18 month longitudinal clinical evaluation by a trained neurologist. The PD cohort was made up of PD controls (*n* = 20), PD (*n* = 20), PDD (*n* = 20) subjects followed and diagnosed via the OPDC. Demographic details for cases and controls in both cohorts are provided within the Supplementary Materials.

For this investigation, the blood collection protocols differed between cohorts. Our approaches to blood collection and specimen processing methods for human investigations have been previously detailed [60,71,72,73,74] and were used for our subacute mTBI specimens. Collection and processing that differed in this study included the lack of fasting for our TBI control group [12], and lack of fasting, medication withholding, and collection of serum rather than plasma in the PD cohort. Input blood specimens for the TBI cohort were collected in ethylenediaminetetraacetic acid (EDTA) tubes, thoroughly mixed, and kept on ice until separated by centrifugation into components (e.g., plasma, leukocytes, erythrocytes), typically within 4 h of collection, except the subacute mTBI group. The latter group’s EDTA tubes were shipped on ice for processing and separation within 24 h of collection, at Georgetown University. Separated plasma was aliquoted into cryovials and placed into −80 °C freezer until analyzed. The PD cohort blood specimens featured collection into BD Vacutainer SSTII tubes. Each tube was mixed via inversion and left at room temperature for 10 min to allow clot formation. Clot tubes then underwent centrifugation, with serum collected into cryovials kept on dry ice until placed into −80 °C freezer for later analyses.

### 4.2. Metabolomic Analyses and Data

For metabolomic analyses utilizing ultraperformance liquid chromatography-mass spectrometry (UPLC-MS), all collected specimens for this study were shipped frozen as individual ≥100 µL aliquots of plasma or serum to the Metabolomics Shared Resource at Georgetown University. All specimens were processed and analyzed using untargeted and targeted methods previously detailed for human studies of preclinical AD [71,73], optimal cognitive aging [74], and acute mTBI [12]. In brief, after sequential extraction [75], untargeted metabolomic profiling of all the plasma specimens was carried out utilizing ultra-performance liquid chromatography-electrospray ionization-quadrupole time of flight-mass spectrometry (UPLC-ESI-QTOF-MS)-based data acquisition and state of the art instrumentation (Acquity H-class UPLC system and Xevo G2 QTOF, Waters Corporation, Milford, MA, USA), with strict adherence to quality control (QC) protocols. Pooled QC samples were run every ten injections. This methodology is conducive to the extraction of a broad range of metabolites, including lipids. Metabolomic relative abundance data output was provided in two ESI modes (negative, NEG, _N; or positive, POS, _P) for each analyzed sample. The Xevo G2 QTOF MS instrument was set up to scan the 50-1200 *m/z* mass range for each ESI mode, for each plasma specimen in the data set. Each ESI mode typically provides up to 3500 unique *m/z* features. The UPLC-MS raw data files were initially pre-processed using the XCMS software [76,77] (Scripps Institute, La Jolla, CA, USA). The untargeted Excel output files produced were populated with mode-specific *m/z* values corresponding to preliminarily annotated metabolites (www.msfmetabolomics.com, [78]) and their relative abundance values within the sample. The untargeted metabolomic approach used in this investigation is considered semi-quantitative [79], and requires an additional step to confirm analyte identification and quantification, typically via tandem mass spectrometry (MS/MS) [80]. The untargeted metabolomic (see Supplementary Material) data in this study has only been preliminarily annotated and has not undergone confirmation of identities via MS/MS. The untargeted metabolomics data were normalized to the intensity of internal standards (debrisoquine in ESI positive and 4, nitro benzoic acid in ESI negative mode) spiked in the extraction buffer. The data are log transformed and Pareto scaled. Targeted metabolomic analysis of plasma/serum samples was performed using the Biocrates Absolute-IDQ P180 (BIOCRATES, Life Science AG, Innsbruck, Austria). This validated targeted assay allows for simultaneous detection and quantification of metabolites in plasma samples (10 L) in a high-throughput manner. The methods have been described in detail [81,82]. The plasma samples were processed as per the instructions by the manufacturer and analyzed on a triple-quadrupole mass spectrometer (Xevo TQ-S, Waters Corporation, Milford, MA, USA) operating in the multiple reaction monitoring (MRM) mode. The measurements were made in a 96-well format for a total of 148 samples, and seven calibration standards and three quality control samples were integrated in the kit. Briefly, the flow injection analysis tandem mass spectrometry (MS/MS) method was used to quantify a panel of 144 lipids simultaneously by multiple reaction monitoring. The other metabolites are resolved on the UPLC and quantified using scheduled MRMs. The kit facilitates absolute quantitation of 21 amino acids, hexose, carnitine, 39 acylcarnitines (ACs), 15 sphingomyelins (SMs), 90 phosphatidylcholines (PCs) and 19 biogenic amines. Pre-analytical processing for the targeted metabolomic data was initially performed using the MetIQ software (BIOCRATES, Life Science AG, Innsbruck, Austria), followed by additional considerations [83], and developed into a similar Excel formatted targeted metabolomic data (see Supplementary Material) as the untargeted metabolomic data.

### 4.3. Metabolomic Biomarker Development

The goal of this analysis was to define a novel metabolomic classifier model that distinguished the subacute mTBI cases from TBI controls and to investigate whether biomarker similarities exist that may implicate TBI in the pathogenesis of PD. A similar untargeted plasma metabolomic biomarker development methodology for the subacute mTBI cohort as was described for our recent human acute mTBI investigation [12], taking advantage of the MetaboAnalyst 4.0 platform (www.metaboanalyst.ca) [84]. The primary steps involved in untargeted biomarker development for this portion of the analyses included running a preliminary annotation of normalized XCMS *m*/*z* features and their respective abundance data for each study subject using the preliminary annotation algorithm MSFmetabolomics (www.msfmetabolomics.com) [78], as previously described [12], and with a mass error stringency of 5 parts per million (ppm). Preliminarily annotated untargeted Excel (.csv) datasets (or similar targeted datasets) were uploaded into MetaboAnalyst 4.0 for biomarker development, utilizing the *Explorer* and *Tester modules*. After initial unbiased multivariate feature selection methods helped define potential biomarker panels, using LinSVM [85], PLS-DA [86], RandFor [87], and LASSO [88] algorithms, a customizable feature selection within the *Tester module* allowed optimization of model results and provided analytic outputs for comparing specific metabolite models via ROC AUC results and the LinSVM, PLS-DA, RandFor, logistic regression (LR) and logistic regression with 10-fold cross validation (LR + 10FCV) methods. 

A second biomarker development approach, via the R package *mixOmics* [89], was used on both the untargeted subacute mTBI cohort metabolomic data as well as untargeted and targeted data from PD/PDD cohort. Complimentary to our prior analyses, we employed these methods to discover and evaluate biomarker signatures discriminating clinical cases (i.e., subacute mTBI or PD/PDD) from their respective healthy controls using both sparse PLS-DA (sPLS-DA) and non-spares PLS-DA methodologies, as appropriate. The sPLS-DA model offers an automated and integrated alternative to the manual selection of variables for inclusion into biomarker panels. Differing from our previous biomarker discovery and statistical work performed on the MetaboAnalyst 4.0 platform, all statistical computing in *mixOmics* was conducted using R. RVU measures initially underwent log base 2 (log_2_) transformation. We also used *mixOmics* to provide ROC AUC results from the targeted metabolomic data derived from the subacute mTBI cohort using sPLS-DA, and to select specific metabolites for modeling using PLS-DA methodology.

### 4.4. Statistical Analyses 

Numerical and categorical comparisons were performed using SPSS Statistics (version 24 for the Mac). Age distributions were plotted to assess normality for case and control groups in each cohort. The comparisons of the two independent group means for age were determined using parametric (*t*-test) and nonparametric (Mann-Whitney U Test) statistics based on normality of age distributions. Categorical analyses for diagnostic group and sex were performed using chi-square analyses. Statistical significance (with Bonferroni correction) was defined at the *p* < 0.025 level. Statistical algorithms within both MetaboAnalyst 4.0 and mixOmics platforms are detailed within their publications [84,89], as previously noted in the Metabolomic Biomarker Development section of the Methods. Both of these platforms utilize feature selection algorithms that account for multiple comparisons inherent in biomarker datasets, where multiple classification features are considered for a relatively small number of specimens (*p* >> *n*). We used ROC AUC results to compare classification of groups and specific biomarker panels in this investigation, with 1.0 indicating error-free classification and 0.5 indicating selection no better than by chance. 

## 5. Conclusions

Based on this preliminary investigation, there appears to be a reciprocal relationship in blood-derived Glutamic Acid levels between cases and controls in our subacute mTBI and PD cohorts. Relatively elevated blood-derived Glutamic Acid was noted in the TBI cases compared to controls, where the opposite was defined in the PD cohort. Although unconfirmed, we propose such a blood biomarker difference may be associated with a central state of glutamate-specific pathobiology. We anticipate that such differences in blood Glutamic Acid levels would be relatively easy to document in a larger number of clinical specimens from similar subject groups and either reproduce or refute this study’s findings. Under optimal conditions such comparisons would be performed on well-matched subject groups and via analysis of a single blood matrix (plasma OR serum) in both cohorts. Although we agree that an ultimate link between TBI and the pathogenesis of PD will require longitudinal assessments of a large number of subjects, future investigations utilizing blood biomarkers and appropriate animal models may provide additional correlative information that may lead to actionable clinical assessments and interventions. Finally, we anticipate that understanding the relationships between blood biomarkers and detailed clinical assessments derived from both TBI and PD subjects will provide additional focus for future investigations, including added neurobiological clues linking these distinct disorders.

## Figures and Tables

**Table 1:** Demographic differences of study cohorts.

Population Characteristic	Subacute TBI Cases	TBI Controls	PD Cases (PD/PDD)	PD Controls
Number of subjects (n)	75	20	40	20
Age in years (mean ± S.D.)	24.9 ± 5.2 *	18.7 ± 0.8 *	67.2 ± 11.4 ^NS^	65.9 ± 10.3 ^NS^
Sex (n; M/F)	71/4 **	8/12 **	22/18 ^NS^	11/9 ^NS^

S.D. = standard deviation. * Statistically significant via Mann-Whitney U test (*p* < 0.025, Bonferroni corrected). ** Statistically significant via chi-square (*p* < 0.025, Bonferroni corrected). NS indicates no significant difference.

**Table 2:** Top 9 common metabolites derived using unbiased feature selection methods.

Preliminary Annotation	RVU in TBI Controls	RVU in Subacute mTBI Cases
* Monoacylglycerol (MG) C16:0_N	Low	High
Taurine_N	Low	High
Sphingosine 1 Phosphate_P (S1P_P)	Low	High
* Glutamic Acid_N	Low	High
**Glucosylceramide (GlcCer) d18:1/26:0_N**	High	Low
* Creatinine_N	High	Low
**GlcCer d18:0/26:0_N**	High	Low
**Phosphatidylcholine (PC) ae C41:1_N**	High	Low
**PC ae C44:5_N**	Low	High

Common metabolites were derived from the top 15 of each feature selection methodology, including linear support vector machine (LinSVM), partial least squares discriminant analysis (PLS-DA), and random forest (RandFor) unbiased algorithms. Comparisons of relative metabolite RVU abundances in TBI controls and cases are presented for each metabolite. * Denotes a top-15 metabolite via the LinSVM, PLS-DA, RandFor, and LASSO feature selection methods. RVU = relative value unit. LASSO = least absolute shrinkage and selection operator. The six metabolites in **bold** combined to provide a convergent logistic regression model. The ae designations for the two PCs indicate that acyl- and alkyl- side chains were represented. Final metabolite identifications will require additional tandem mass spectrometry (MS/MS) analyses. Metabolites confirmed via MS/MS are considered fully validated, to a high degree of confidence.

**Table 3:** Classification results for the convergent 6-metabolite subacute mTBI panel.

Classification Algorithm for Model	ROC AUC	95% CI	Sensitivity/Specificity
LinSVM	0.968	0.945–0.992	-
PLS-DA	0.977	0.945–0.992	-
RandFor	0.965	0.882–1.00	-
LR	0.939	0.734–0.984	-
LR + 10FCV Discovery	0.993	0.984–1.00	0.981/0.939
LR + 10FCV Internal Validation	0.893	0.789–0.996	0.947/0.850

mTBI = mild traumatic brain injury. ROC AUC = receiver operating characteristic area under the curve. CI = confidence interval. LinSVM = linear support vector machine. PLS-DA = partial least squares discriminant analysis. RandFor = random forests. LR = logistic regression. LR + 10FCV = logistic regression with 10-fold cross validation.

## Supplementary Materials

The following are available online at http://www.mdpi.com/2218-1989/8/3/50/s1, Supplementary READ ME file 1; S2, Supplementary targeted metabolomic Excel files (1–4); S3, Supplementary untargeted metabolomics Excel files (1,2); S4, Supplementary demographics Excel file.
