# Supplementary material for: Potential Metabolomic Linkage in Blood between Parkinson’s Disease and Traumatic Brain Injury
Source: Metabolites. 2018 Sep 7;8(3):50. doi: 10.3390/metabo8030050 (PMC6161135; doi:10.3390/metabo8030050)
Supplement: Supplementary file 1 [file metabolites-08-00050-s001.zip › 1.Supplementary Material READ ME Document.docx]

**Supplementary Material Document**

READ ME

The following data are planned for inclusion in the Dryad Digital Repository associated with the publication by Fiandaca et al, titled **Potential Metabolomic Linkage in Blood Between Parkinson’s Disease and Traumatic Brain Injury** to be included in the *Metabolites* Special Issue: Metabolomics in Neurodegenerative Disease (Edited by Dr. Brian Green).

**1. Supplementary Material READ ME Document.docx**: which you are currently reviewing, provides a list of all supplementary files to the manuscript. (**144 KB**)

**2.** ***Metabolites* Supporting Info Archive 1.zip (329 KB)**: containing targeted metabolomic Excel worksheet files from the subacute mTBI Cohort run on the R package, *mixOmics*, as described in the manuscript. Includes Set 1: Runs 1 (**283 KB**) & 2 (**282 KB**); and Set 2: Runs 1 (**211 KB**) & 2 (**211 KB**).

**3. *Metabolites* Supporting Info Archive 2.zip (19.2 MB)**:

**A.** **Untargeted Subacute mTBI Cohort.xlsx** **(10.9 MB)** - Excel worksheet (and 13 tabs) of normalized XCMS data from the subacute mTBI Cohort used in biomarker development strategies described in the manuscript, taking advantage of the MetaboAnalyst 4.0 online analytic platform ([www.metaboanalyst.ca](http://www.metaboanalyst.ca)). Tabs are numbered (1-13) and labeled to indicate specific data processing steps from original XCMS (tabs 2 & 3) to finalized data (tab 13) prior to conversion to two-group comparison .csv files used within MetaboAnalyst 4.0. Note that preliminary annotation outputs are provided in tabs 4-7, using MSFmetabolomics.com.

**B.** **Untargeted PD Cohort.xlsx (8.3 MB)** - Excel worksheet (and 14 tabs) of normalized XCMS data from the PD Cohort used in biomarker development strategies described in the manuscript, taking advantage of the MetaboAnalyst 4.0 online analytic platform ([www.metaboanalyst.ca](http://www.metaboanalyst.ca)). Tabs are numbered (1-14) and labeled to indicate specific data processing steps from original XCMS (tabs 2 & 3) to the finalized data tab (14) prior to conversion to two-group comparison .csv files used within MetaboAnalyst 4.0. Note that preliminary annotation outputs are provided in tabs 4-7, using MSFmetabolomics.com.

**4.** ***Metabolites* Cohorts Demographics.xlsx (66 KB)**: Excel file information for each of the groups making up the two study Cohorts. Three tabs provide information for the TBI Control group (n=20) included in the subacute mTBI Cohort, the NHCL Subacute TBI group (n=75) from the TBI Cohort, and the OPDC PD Cohort (n=60).
